# A Review of Phytochemical and Pharmacological Studies on *Galium verum* L., Rubiaceae

**DOI:** 10.3390/molecules30081856

**Published:** 2025-04-21

**Authors:** Margarita Koleva Petkova, Neli Hristova Grozeva, Milena Tankova Tzanova, Mima Hristova Todorova

**Affiliations:** 1 Department of Biological Sciences, Faculty of Agriculture, Trakia University, 6000 Stara Zagora, Bulgaria; milena.tsanova@trakia-uni.bg; 2 Department of Plant Breeding, Faculty of Agriculture, Trakia University, 6000 Stara Zagora, Bulgaria; mima.todorova@trakia-uni.bg

**Keywords:** antioxidants, biological activity, flavonoids, *Galium verum* L., Rubiaceae, iridoids, pharmacology, phenols, phytochemistry

## Abstract

*Galium verum* (Yellow Bedstraw) is a rhizomatous perennial herb belonging to the Rubiaceae family. It is native to Eurasia and Africa but has also been introduced to southern Canada and the northern U.S. Widely used in traditional medicine, *G. verum* has been recognized for its diuretic, anti-inflammatory, antimicrobial, analgesic, and anticancer properties. Phytochemical studies have shown that the plant is rich in significant bioactive compounds, such as flavonoids, phenolic acids, iridoids, anthraquinones, phytosterols, coumarins, and tannins. Research suggests that *G. verum* exhibits strong antioxidant activity, protecting cells from oxidative stress and inflammation. Its antimicrobial potential has been demonstrated against various bacterial and fungal pathogens, supporting its traditional use in wound healing and infection treatment. Moreover, modern studies indicate its cytotoxic effects on cancer cells, suggesting potential applications in oncology. Additionally, its hepatoprotective and neuroprotective properties highlight its promise for treating metabolic and neurodegenerative disorders. Despite its well-known therapeutic potential, further studies are required to fully clarify its mechanisms of action and ensure its safety for medicinal use. Given the variety of bioactive compounds found in *G. verum* and their pharmacological benefits, this review emphasizes the importance of this species as a valuable medicinal plant, encouraging further scientific research for its application in pharmacology.

## 1. Introduction

In official and traditional medicine, various medicinal plants have been used from antiquity for disease prevention and therapeutic purposes [1]. Plants are a natural source of bioactive substances, many of which have beneficial pharmacological effects for humans [2]. According to Delprete et al., the Rubiaceae family is one of the largest angiosperm families with a large number of species distributed throughout the world, including deserts and high mountains [3]. The overall phytochemical composition of Rubiaceae plants lends to their potential as sources of bioactive compounds [4]. This family has attracted significant attention from researchers due to its diverse pharmacological applications. Many studies detect flavonoids, iridoids, triterpenes, phytosterols, anthraquinones, and others. Heitzman et al. claimed that plants in the Rubiaceae family are widely used in traditional medicine for their diverse therapeutic effects, including antioxidant, antiviral, antibacterial, and anti-inflammatory properties [5]. Additionally, these plants exhibit activity against cardiovascular and central nervous system diseases. The Rubiaceae family also has significant economic importance. For instance, species in the Ixoroideae subfamily are rich in caffeine, a compound that acts as a stimulant for the central nervous system, as well as a diuretic, bronchodilator, and antimigraine agent, as highlighted by Martins et al. [6]. Quinine, a compound isolated from *Cinchona calisaya* Wedd. in the Cinchonoideae subfamily, was the only antimalarial drug for over 200 years, and it has the highest quinine concentrations identified to date [7,8]. The discovery of quinine was revolutionary as it also paved the way for the development of synthetic drugs to treat malaria, which greatly reduced the mortality caused by malaria. The use and importance of natural quinine is still significant and even increasing [9].

Belonging to the Rubiacaeae family and the Rubioideae subfamily is the genus *Galium*, also known as *Bedstraw,* which is pharmacologically significant and is the most species-rich genus within the tribe Rubieae [10,11,12].

The genus *Galium* comprises approximately 667 species globally, with one-third occurring in Europe [13,14]. Furthermore, approximately 300 species within this genus have been identified as having medicinal properties [5]. These activities are attributed to their rich content of bioactive compounds such as phenolic compounds (including flavonoids) [15,16,17,18,19,20,21,22,23,24,25,26,27,28,29,30,31,32,33,34,35,36,37,38,39,40,41], phenolic acids [16,22,23,25,35], iridoids [42,43,44,45,46,47,48,49,50], anthraquinones [41,51,52,53,54], terpenoids (including saponins) [33,47,55,56,57,58,59], yellow pigments [52], essential oils [56,58], vitamin C [19,60], phytosterols [61,62], and enzymes [63].

The species of the genus *Galium* possess a wide range of biological activities, including immunomodulatory [32,64], anticancer [65,66], antihaemolytic [67], hepatoprotective [68], diuretic [69], antioxidant [15,23,31,39,70,71,72,73], apoptotic [74,75,76], anti-inflammatory [38,40,77,78], sedative [79], astringent [35,79], wound healing [73,80], enzyme activating [81], and antimicrobial effects [23,73,82,83,84].

*Galium verum* L. (commonly known as Yellow or Lady’s Bedstraw or Eniovche in Bulgarian) is one of the most widespread species of the genus *Galium*. It is rich in phytochemical compounds with significant biological activity, making it one of the most versatile medicinal plants traditionally used for the prevention and treatment of many diseases due to its anti-inflammatory, anticancer, hepatoprotective, diuretic, cardioprotective, and astringent properties [21,35,65,66,67,68,69,70,71,72,75,76,77,79,80,81,82,83,84,85,86]. This species has deep roots in Bulgarian traditional medicine, and there is even a holiday named after it, celebrated every year on June 24th. Its name is Eniovden, and it is also known as Midsummer—the longest day of the year. On this day, it is believed that the herbs have the greatest power, and according to tradition, women should go out early in the morning to collect them [87].

The aim of this review is to provide an objective assessment of the available data on the phytochemical composition of *Galium* species, particularly *G. verum,* and the pharmacological effects of the identified classes of metabolites. The goal is to highlight the therapeutic benefits of this medicinal plant and to achieve a more comprehensive understanding of the genus. Such efforts will help identify areas where further research is needed, stimulate more in-depth clinical trials, and potentially lead to the discovery of new pharmacological effects. The final result suggests the rational utilization of *Galium* species as a valuable source of bioactive substances.

## 2. Materials and Methods

This review was compiled using a comprehensive search strategy that included collecting data from journals and books published between 1971 and 2025, sourced from databases such as EBSCOhost, Web of Science, ScienceDirect, Scopus, Scimago Journal, PubMed, PubChem, ResearchGate, and Elsevier. The search criteria aimed to identify studies that reported quantitative and qualitative determination and biological activity of phytochemical compounds derived from *Galium* species. Studies on the mechanisms of action of phytochemicals that have been proven to be present in *Galium* species but have not been specifically studied in them were also included. Inclusion criteria involved articles related to the phytochemistry of *Galium* species, along with the corresponding biological and pharmacological activities. Exclusion criteria comprised studies that lacked experimental confirmation and chemical characterization.

The two-dimensional chemical structures included in this review were prepared using the software package Revvity ChemDraw, version 23.1.2 (PerkinElmer Informatics, 2023).

The phytochemical compounds included in this review were selected, as they are the most frequently studied in *Galium* species by researchers and are proven to affect organisms at the cellular level through the mechanisms of biological activities they exhibit. A tabular format was chosen to summarize the information obtained, making it easily accessible and understandable.

## 3. Results and Discussion

### 3.1. Phytochemical Composition and Biological Activity of Galium Species

The pharmacological properties of Rubiaceae plants are largely attributed to the content of various phytochemical compounds with diverse biological effects. While these effects are of significant scientific interest, they remain incompletely researched.

The genus *Galium* has been used in traditional medicine for ages due to its therapeutic properties [5,77,79,80,82].

Many studies have focused on the potential of specific *Galium* species, known for their therapeutic properties in ethnomedicine, such as diuretic, sedative, wound healing, and immunomodulatory [73,79,80,81]. Similar compounds are detected within the genus, which often demonstrate potent biological activity, determining their mechanisms of action in biological systems. Table 1 summarizes the presence of different phytochemical groups of compounds in some *Galium* species.

As presented in Table 1 the phytochemical composition of the *Galium* genus varies significantly among species; however, flavonoids and iridoids are the most commonly reported bioactive compounds. Aerial parts of the plants are richer in flavonoids, while the roots contain higher concentrations of iridoids [28].

*Galium verum* and *G. aparine* were found to be the richest in flavonoids, such as quercetin, rutin, and luteolin, while *G. mollugo* and *G. odoratum* contained more kaempferol and diosmetin [17,19,28,62].

The high content of flavonoids and polyphenols correlates with the strong antioxidant activity of *Galium* species [19,20,22,26,40,78,88], which determine their anticancer [84], immunomodulatory [31], and antimicrobial effects [25,73].

Iridoid compounds are isolated from many *Galium* species and are known to contribute to antioxidant, anti-inflammatory, and anticancer properties of plant-derived products [23,30,37,89,90]. Asperuloside is the most consistently found iridoid across all species of the genus, with *G. verum* and *G. mollugo* being the richest sources [43,44,45,46,47]. Other iridoids like monotropein, loganin, and geniposidic acid are more species specific and are used for taxonomic markers [46].

Anthraquinones found in *Galium* species exhibit antimicrobial and anticancer properties [6,54]. Phytosterols help reduce blood cholesterol and exhibit antitumor activity [54,61,62]. Saponins exhibit antifungal and antimicrobial potential [55,57].

Most widely used in modern and traditional medicine for their antioxidant, antibacterial, antifungal, and antiparasitic properties are *G. verum*, *G. aparine*, *G. mollugo*, and *G. odoratum* [28,91].

*Galium verum* blossoms and herb, *G. aparine* herb, and *G. mollugo* herb are a valuable source of phenolics, iridoids, and volatile phytochemicals long known to mankind for their therapeutic properties [28,37].

Natural products of *G. aparine* such as flavonoids, terpenoids, and steroids also have different pharmacological properties, including antioxidant and antitumor activity [74]. As a natural antioxidant *G. aparine* is used in the treatment of various diseases such as diabetes, cancer, and hypertension [92].

By virtue of their antioxidant properties, *G. verum* and *G. aparine* are traditionally used in Europe and the Balkans as diuretics, blood purifiers, and liver detoxifiers, consumed as tea for kidney and liver disorders and urinary tract infections. Externally, they are applied as wound healers for eczema, ulcers, and burns [28,32,33,35,69,73,77,79,93]. Antioxidant properties of *Galium* species are also utilized in derma cosmetics [14].

*Galium mollugo* has moderate antioxidant activity and exhibits antibacterial properties [33,46]. It is externally used for skin irritations and inflammations as it showed strong anti-inflammatory potential, likely due to its high iridoid content, and it is internally used as a diuretic and digestive aid [33,46].

Due to its antioxidant, neuroprotective, and anti-inflammatory properties, *G. odoratum* is recognized for antispasmodic, sedative, and anticoagulant effects and is used for digestive support [23,33,46,62,93].

### 3.2. Phytochemical Composition, Biological Activity, and Pharmacological Effects of G. verum

*Galium verum* is one of the most significant herbs in ethnomedicine and has recently attracted the attention of scientists for its diverse biological activities and its rich phytochemical profile. Research on the phytochemistry of *G. verum* supports its traditional use and potential medicinal application. The latest studies on the composition of the species overlap with older ones, with some documenting the content of substances unknown to the species. As a source of phenolic compounds, such as flavonoids and phenolic acids, iridoids, anthraquinones, and other bioactive compounds, *G. verum* has been used for its antioxidant [33,34,37], anti-inflammatory [38,54,71,72], immunomodulatory [32], diuretic [69,79,80], sedative [79,82], wound healing [35,80], anticancer [65,66,84], antiseptic [82], and antibacterial [84] properties for treating kidney and liver diseases [33] and cardiovascular diseases [86], and it is applied externally for healing wounds, ulcers, and rashes [35,80,82].

The phytochemical compounds isolated from *G. verum* and determined, and their biological activities (where information is available) are illustrated in Table 2.

As shown in Table 2. numerous phytochemical compounds have been reported in the content of *G. verum*. Many of them correlate with certain biological activity. To understand how these compounds contribute to the healing properties of the species, a more detailed review of the available research for each group of bioactive compounds and the correlating pharmacological effects will be discussed in the subsections below.

#### 3.2.1. Content of Phenolic Compounds (Flavones, Flavonols, Flavone Glycosides, Flavonol Glycosides, Phenolic Acids) and Antioxidant Activity

The extraction of phenolic compounds from medicinal plants has attracted growing interest due to their significant antioxidant properties and widespread presence in plants [94,95,96]. Antioxidants have protective roles against the harmful effects of various drugs and chemicals. They can mitigate oxidative stress, a key factor in cellular damage, thereby offering potential therapeutic strategies to counteract toxicity induced by pharmaceuticals and environmental agents [97]. They also have the potential to mitigate the effects of oxidative stress in aging and related diseases [98].

The antioxidant activity of *G. verum* mainly relies on its rich phenolic content. Polyphenols possess potent antioxidant properties, modulating enzyme and cellular receptor activity, regulating nitric oxide levels, inducing apoptosis, scavenging free radicals, and inhibiting cell proliferation and angiogenesis [99,100,101,102]. These mechanisms underline their preventive effects, including anti-inflammatory, antimicrobial, cardioprotective, and other health-promoting activities. A number of epidemiological data indicate that these activities may be effective against neurodegenerative diseases, cardiovascular conditions, and disorders linked to oxidative stress [103,104,105].

Mavi et al. were among the first to examine the antioxidant activity of *G. verum* [15]. Using various methods, a connection between the reducing power of plant extracts and the total phenolic content was identified. As extract concentrations increased, so did their phenolic content, reducing power. Despite a relatively low total phenolic content detected, *G. verum* exhibited strong antioxidant activity, including DPPH (2,2-diphenyl-1-picrylhydrazyl) radical scavenging activity and peroxidation inhibition, suggesting that its phenolic compounds are powerful antioxidants.

Tămaş et al. conducted comparative phytochemical research of *G. verum L*. and *G. mollugo* L. [16]. Using TLC, it was established that these species contained five flavonoid compounds: hyperoside (12), quercitrin (20), rutin (21), caffeic (22), and chlorogenic acid (23). The amounts of the identified compounds were different for the species studied. *G. verum* contained higher amounts of hyperoside, while *G. molugo* contained higher amounts of rutin. The total content of flavonoids was also different, with over three times higher amounts for *G. verum*.

Zhao et al. conducted a detailed analysis of the phenolic composition of *G. verum* [17,18]. Their research identified several phenolic compounds, including diosmetin (8), isorhamnetin (14), kaempferol (17), and quercetin (19).

Lakić et al. conducted a comparative study of two *G. verum* populations in Serbia and found significant differences in flavonoid content, chlorophyll levels, and radical-scavenging activity based on geographical and environmental factors [19]. The following indicators were determined in the study: total phenolic and flavonoid content; chlorophyll “a” and “b” content; and antioxidant activity by several methods: DPPH radical scavenging activity, OH radical scavenging activity, H_2_O_2_ scavenging activity, and lipid peroxidation. The researcher found higher values of phenolic content (4.57–5.16 mg GAE/g dm) (GAEs—gallic acid equivalents), flavonoids, and quercetin equivalents (15.56–17.96 μg QE/g dm) in the population exposed to environmental urban stressors, and on the contrary, higher total content of chlorophyll “a” and “b” in plants from the mountain populations (0.25 mg—chlorophyll “a”; 0.46 mg—chlorophyll “b”), which are exposed to direct sunlight at higher altitudes. This corresponds to the higher IC_50_ values of the DPPH analysis: 8.04 µg/mL for the urban population vs. 3.10 µg/mL for the mountain population. The authors observed a stronger dependence of DPPH radical-scavenging activity on the extract concentration. The same tendencies were determined at the hydroxyl radical scavenging tests. IC_50_ values of 0.05 µg/mL were determined for the samples from the mountain population and 0.54 µg/mL for the urban samples. Reversed, the degree of neutralization of a 50% solution of hydrogen peroxide (H_2_O_2_) varies from 4.98 µg/mL to 3.80 µg/mL for the mountain and urban samples, respectively. More general conclusions were drawn in the same work. The degree of inhibition of lipid peroxidation varied widely, ranging from 2.07% to 69.39% and from 23.91% to 48.25% for the mountain and urban samples, respectively. Both tested extracts expressed a noticeable inhibitory activity on lipid peroxidation, with the following values established for IC_50_: 11.70 µg/mL and 19.47 µg/mL, respectively. Lakić et al. concluded that the superior neutralization of DPPH and hydroxyl radicals by mountain plant extracts was due to higher chlorophyll content, while the better hydrogen peroxide scavenging observed in Veternik extracts correlated with higher phenolic and flavonoid levels.

Data from analysis of ethanol extracts from *G. verum* var. *asiaticum* from Korea showed strong antioxidant activity [20]. The researcher explained it by the high content of phenolic compounds such as luteolin (18), rutin (21), and caffeic acid (22). Caffeic acid was identified as the compound with the most significant antioxidant activity.

Zhao et al. discovered that diosmetin (8) isolated from *G. verum* exhibits an anticancer effect [21]. Treated mice with this compound developed a dose-dependent effect on tumor growth, and the degree of inhibition increased with the dose increasing. Diosmetin application protected cells from tumor-induced apoptosis.

Danila et al. conducted a comparative analysis of several medicinal plants, including *G. verum,* from the territory of Romania [22]. The study determined the total phenolic content via spectrophotometry and identified the phenolic compounds’ high-performance liquid chromatography (RP-HPLC). Antioxidant activity was determined by DPPH radical scavenging activity and ABTS (2,2′-azino-bis(3-ethylbenzothiazoline-6-sulfonic acid) cation radical scavenging activity. The results showed relatively high total phenolic content and antioxidant activity for *G. verum*. Alcoholic extracts yielded higher phenolic concentrations than aqueous ones, with optimal yields for *G. verum* obtained using 30% ethanol. This approach of comparing the type of extract highlighted the differences between the concentrations of phenolic compounds as a function of the extraction solvent. To determine the phenolic and flavonoid composition of the analyzed plants, the authors developed and validated a new HPLC method. The following phenols were identified: caffeic acid (22), chlorogenic acid (23), coumarinic acid (24), and ferulic acid (26), and the flavonoids kaempferol (17), luteolin (18), quercetin (19), and rutin (21). Chlorogenic acid was the predominant compound, followed by caffeic and coumarinic acids. The researchers concluded that the high phenolic content and antioxidant activity of *G. verum* make it a promising candidate for medical purposes.

Vlase et al. conducted a comparative study of four *Galium* species collected from Romania [23]. Polyphenol and flavonoid content, antioxidant activity, and antimicrobial properties of 70% ethanol extracts further reinforce the medicinal potential of *G. verum*. Using the HPLC-MS/MS method, diverse polyphenolic compounds were identified in *G. verum* extracts, some of which had been previously reported for this species: isoquercitrin (16), kaempferol (17), quercetin (19), quercitrin (20), rutin (21), chlorogenic acid (23), p-coumaric acid (25), and ferulic acid (26). Among these, chlorogenic and ferulic acids were present in minor quantities, while rutin was identified as the predominant compound, with a concentration of 804 mg/100 g of dried plant material. Interestingly, compounds, such as luteolin, caffeic acid, and apigenin, which were identified in prior studies, were not detected in this analysis. This suggests that the polyphenol composition may vary across populations, influenced by ecological factors such as habitat, altitude, and latitude. Additionally, variations may arise from differences in the identification methods, solvents, or extraction techniques used (e.g., with or without ultrasound). In the same study, the total content of polyphenols, flavonoids, and caffeic acid derivatives was quantified using spectrophotometric methods. Among the four species studied, *G. mollugo* and *G. verum* exhibited the highest levels of these phytochemical compounds. Based on these findings, Vlase et al. suggested that both species hold potential as sources of antioxidants for pharmaceutical applications. Researchers supported this claim with additional studies. Antioxidant activity was measured using the DPPH radical scavenging assay, and the results connected strongly with the total phenolic content. *Galium verum* demonstrated the highest antioxidant potential among the species studied, with an IC_50_ value of 105.43 ± 0.15 µg/mL. The antibacterial activity of the extracts was evaluated using a diffusimetric method. The results indicated varying degrees of inhibitory activity against the tested pathogens, particularly against Gram-positive bacteria, such as *S. aureus* and *L. monocytogenes*. *G. verum* exhibited moderate antibacterial activity but showed limited antifungal activity against *C. albicans*. The comparative study by Vlase et al. highlighted significant differences in the polyphenol content among the four *Galium* species, suggesting that these differences could serve as taxonomic markers for distinguishing species within the genus [23].

The pro-oxidant reactivity of the extracts was evaluated by Moţ et al. [24]. The reactivity was significantly higher than the average for other plant extracts tested with the same method. This increase was attributed to the quercetin present in the *G. verum* extract, which is known for its ability to generate radicals easily. The second stage of the study included an in vitro experiment with female rats. They were exposed to oxidative stress by being kept in complete darkness. The rats treated with *G. verum* extracts showed reduced oxidative damage and restored antioxidant balance compared to untreated groups. This study demonstrated that *G. verum* extract may have the potential to counteract biochemical damage caused by stress and highlighted *G. verum*’s significant potential as a source of antioxidants. Thus, it could be considered for use in pharmaceutical formulations, although further research is necessary to confirm these findings.

Matei et al. conducted further phytochemical studies on *G. verum* and related species [25]. They found that *G. verum* contains a 3-fold higher flavonoid concentration than other species, which explains its notable antioxidant activity. A total of 13 phenolic compounds were identified, including catechin (4), chrysin (5), epicatechin (9), fisetin (10), hesperidin (11), isorhamnetin (14), isoquercitrin (16), quercitrin (20), rutin (21), caffeic acid (22), chlorogenic acid (23), p-coumaric acid (25), and ferulic acid (26). The study developed an HPLC method to quantitatively analyze these compounds in ethanol extracts. It was observed that the type of solvent and extraction method significantly influenced the results, with reflux extraction yielding the most efficient results.

Layali et al. focused their research exclusively on *G. verum* to evaluate its potential for use in traditional medicine and as a functional food source [26]. Plants were collected from Iran, and extracts from the aerial parts were analyzed using four different in vitro systems to measure radical scavenging activity. These methods included DPPH radical scavenging activity, nitric oxide scavenging activity, hydrogen peroxide scavenging rate, and iron-reducing power (rate of conversion of Fe(III) to Fe(II) by electron donation), and the results obtained by another researcher were confirmed [22]. The authors confirmed the well-documented antioxidant potential of flavonoids, attributing their efficacy to the hydroxyl groups responsible for free radical scavenging. These results emphasize the potential health benefits of increasing flavonoid consumption in the diet, as supported by previous studies.

Total phenolic and total flavonoid content in *G. verum* was determined by classical colorimetric methods, developed earlier by Ebrahimzadeh [106,107], and the results obtained were 753 ± 21 mg GAE/g dm and 151.25 ± 8.2 mg QE/g dm. These high values underscore *G. verum*’s potential as a significant source of phenols. DPPH radical scavenging activity increased with higher extract concentrations, yielding an IC_50_ value of 59.6 ± 0.04 µg/mL. For comparison, ascorbic acid and BHA (butylated hydroxyanisole) had IC_50_ values of 8.78 ± 0.21 and 92.9 ± 4.5 µg/mL, respectively. Nitric oxide scavenging activity was exceptionally high, with an IC_50_ of 1.7 ± 0.01 µg/mL, and the extract demonstrated a 92.5% inhibition rate of H_2_O_2_ at a concentration of 50 µg/mL. Additionally, the extract showed strong iron-reducing capabilities at concentrations of 50–800 µg/mL, comparable to vitamin C at higher concentrations (*p* > 0.05).

Dong et al. further validated the protective effects of flavones derived from *G. verum* using human umbilical vein endothelial cells subjected to oxidative stress induced by hydrogen peroxide [27]. The flavones significantly reduced oxidative damage by enhancing nitric oxide levels. A different approach was taken in the study of Ilyina et al., who explored the relationship between the morphological characteristics of *G. verum* and its flavonoid content [28]. The study revealed correlations between specific morphological traits and the chemical profiles of the species.

The research by Cheng et al. was in a different direction [29]. This research proved the anti-thrombotic effect of diosmin (the aglycone part of diosmetin) extracted from *G. verum* by treating rats. The depletion of the key protein for the prevention of thrombosis, CER350, reduces the stability of microtubules. During the experiments, it was found that diosmin (7) causes protein changes and prevents the protein levels from dropping.

Kuhtinskaja et al. compared four extraction methods for bioactive compounds from two plant species, including *G. verum* [30]. The results did not show a clear advantage of one method. Chlorogenic acid (23) was most effectively extracted from *G. verum* with pressurized hot water, whereas rutin (21) was most efficiently extracted using methanol. Despite slight variations, all tested methods were deemed effective for extracting phenols and iridoids, with hot water extraction being preferred due to its environmental friendliness. In the same study, using HPLC- MS (HPLC with mass detection) identified 14 major compounds in *G. verum* extracts, including flavonols and iridoid glucosides, reaffirming the species’ rich phenolic and iridoid content.

In 2018, Farcas et al. used in vitro analyses to evaluate the antioxidant and pro-oxidant activities of the flavonoids contained in *G. verum* [31]. The first stage of the study determined the phytochemical composition of *G. verum* using HPLC. High levels of rutin (21) and chlorogenic acid (23) were identified, confirming their role as anti-stress modulators. The antioxidant activity of the extracts was assessed using the DPPH and TEAC methods (Trolox-equivalent antioxidant capacity), with DPPH showing higher activity levels.

Shynkovenko et al. investigated the phytochemical profile of *G. verum*, with results that were largely consistent with previous studies [32]. Any differences were likely due to variations in habitat conditions and analysis methods. The researchers also examined the immunomodulatory effects of the ethanolic extract of *G. verum* using an in vitro experiment with lymphocytes (immune system cells) from heparinized blood. The study is the first of its kind for *G. verum*. It was found that all the substances tested exhibited a significant stimulating effect on the transformation activity of peripheral blood mononuclear cells. The highest immunomodulatory activity was observed with the 96% ethanol extract, which increased proliferation compared to spontaneous transformation. These findings provide a foundation for further research into the immunomodulatory activity of *G. verum* extracts.

Friščić et al. studied the bioactive components and antioxidant potential of eight species of the genus *Galium* in Croatia [33]. Qualitative phytochemical analysis, spectrophotometric determination of total phenols, flavonoids, and iridoids, and analysis of antioxidant activity using the ABTS and DPPH tests were carried out. The results revealed that *G. verum* contains some of the highest levels of total phenolics (86.40 ± 1.74 mg GAE/g dm) and flavonoids (23.11 ± 0.12 mg QE/g dm) and demonstrated strong antioxidant activity. The presence of other bioactive substances further suggests biomedical potential, warranting further in-depth studies.

In the same year, Hanganu et al. published data on three species of the genus *Galium* from Romania [34]. The study was similar to Friščić’s study, but different methods of analysis were used. Extraction was performed with 70% ethanol, and antioxidant activity was assessed using the FRAP (ferric ion-reducing antioxidant power) method (using Trolox), the CUPRAC method, and the xanthine oxidase method. The study showed that *G. verum* had the broadest spectrum of extracted chemical compounds, although *G. odoratum* had higher levels of total phenols and flavonoids. All three extracts exhibited strong antioxidant capacity, which was directly correlated with their total phenol and flavonoid content. These findings align with most of the available literature, except for the study by Vlase et al. [23], which reported that *G. verum* had higher flavonoid content and antioxidant potential compared to *G. aparine*.

Vuletic et al. assessed the medicinal properties of *G. verum* in an in vivo experiment with rats [35]. In the first phase of the study, the phytochemical composition of the species was analyzed by the HPLC-DAD (HPLC with diode-array detection) technique. In the second phase, a mucoadhesive gel based on *G. verum* extract was developed and applied to rats, with recurrent aphthous stomatitis induced in a laboratory setting. Similar to previous studies on *G. verum*’s phenolic composition, quercetin (19), quercitrin (20), rutin (21), caffeic acid (22), chlorogenic acid (23), p-coumaric acid (25), and gallic acid (27) were identified. Rutin had the highest amount of 23.81 ± 1.90 mg/g dm. For the in vivo experiment, 60 male rats were used. Ulcers were induced in their oral cavity by applying glacial acetic acid. The rats were divided into three groups: Group 1—negative control (no treatment), Group 2—control treated with a gel base without *G. verum* extract, and Group 3—test group treated with a 20% *G. verum* gel. By the second day of gel application, the test group showed significantly more effective ulcer shrinkage compared to the control groups. This improvement continued throughout the experiment, with the test group achieving 100% ulcer shrinkage by the 6th day, which was twice the shrinkage percentage seen in the control groups. In conclusion, the researchers summarized that treatment with *G. verum* reduced pro-oxidant generation in tissues, which helped limit oxidative stress and accelerated the healing process. These effects are likely due to the synergistic activity of the phenolic compounds present in the *G. verum* extract.

In 2023, Ohindovschi et al. used TLC to identify the phenolic content of hydro-ethanol plant extract obtained from *G. verum* [36]. The chromatographic study revealed the content of apigenin (1), hyperoside (12), isoquercetin (15), quercetin (19), quercitrin (20), and rutin (21), and the following phenolic acids: caffeic (22), chlorogenic (23), p-coumaric (25), and gallic (27).

In the same year, Laanet et al. conducted a study on three species of the genus *Galium*, including *G. verum*, from Estonia [37]. The analysis focused on the content of volatile and non-volatile chemical compounds and antioxidant activity. The sensitive HPLC-MS method and colorimetric tests were used for phytochemical analysis. For antioxidant capacity assessment, the ORAC (Oxygen Radical Absorbance Capacity) method was applied [108]. Volatile compounds were quantified by SPME-GC-MS (solid-phase microextraction with gas chromatography with mass detection). Hexanal, anethole, and β-caryophyllene (≥1%) were detected in all samples. The results from colorimetric tests showed that *G. verum* has high levels of polyphenols (up to 27.2 ± 1.5 mg GAE/g), flavonoids (up to 7.3 ± 0.5 mgQE/g), and iridoids (up to 40.8 ± 2.9 mgEq asperuloside/g). Chromatographic analyses identified individual chemical compounds, revealing differences between the flowers and the whole plant. The flowers contained higher concentrations of certain compounds, with the highest content of asperuloside (28) found in both flower and whole stems. The analysis of antioxidant activity showed that all the studied species exhibit a strong antioxidant capacity (up to 9.3 ± 1.2 mgTE/g). Among the studied species, extracts from *G. verum* flowers stand out for containing the highest amounts of bioactive substances and exhibiting the most pronounced antioxidant potential. The polyphenolic composition is dominated by rutin (21) and chlorogenic acid (23), both known for their antioxidant properties. The rutin content in *G. verum* is the highest among the studied species. The results of the analyses conducted by the researchers show a clear correlation between the total polyphenol content and the antioxidant activity of *G. verum* extracts. The antioxidant properties of plant extracts from the genus *Galium* reveal their potential as future therapeutic agents for treating diseases caused by oxidative stress. Further in-depth studies are necessary to elucidate their mechanisms of action, optimize extraction methods, and assess their clinical efficacy. This will contribute to the development of novel therapies using the antioxidant effect of plant-derived products.

Antoniak et al. studied the antiangiogenic and anti-inflammatory properties of several medicinal plant species, including *G. verum* [38]. The researchers used an in vitro approach, applying ethanol extracts from various species of the genus *Galium*, which are rich in bioactive substances with potential antiangiogenic effects, to endothelial cells from human umbilical cord blood vessels in a controlled environment. They aimed to evaluate the effect of plant extracts on endothelial cell viability, cell proliferation, migration, invasiveness, and the production of angiogenic and angiostatic factors. Prior to the in vitro experiment, the following analyses were conducted: total phenolic content, volatile compounds content determined by GC-MS, and antioxidant activity by the DPPH and FRAP methods. The results for *G. verum* from the spectrophotometric study showed a high phenolic content and fresh weight (58.47 ± 4.40 mg GAE/g FW). Five different volatile compounds in the plant extract (7-ethyl-4-decen-6-one, ascaridole epoxide, 4-hydroxy-benzenepropanoic acid, (E,Z,Z)-2,4,7-tridecatrienal, and estra-1,3,5(10)-trien-17ß-ol) were identified. The tests confirmed the antioxidant properties of all three species studied, indicating that these properties correlate with the high polyphenol content. In vitro experiments showed that ethanolic plant extracts of all three species significantly increased cell invasiveness compared to the control group. However, only two species, including *G. verum*, reduced cell proliferation. These species also exhibited the most pronounced anti-inflammatory activity. Furthermore, the tested extracts reduced the production of proangiogenic platelet-derived growth factor (PDGF) and hepatocyte growth factor (HGF) and thus may affect angiogenesis at various levels. In conclusion, the authors highlighted that the modifying effects of the studied extracts could have beneficial implications for pathological processes related to free radical formation, inflammation, and angiogenesis.

Rashed et al. performed an experiment with rats that demonstrated the antioxidant effect of *G. verum* [39]. The rats were subjected to acute kidney injury, induced by folic acid, and divided into groups. One of the groups was treated with *G. verum* extract orally for seven consecutive days. The results showed improvement in kidney structure and enhanced renal function recovery in the treated group compared to the other groups.

Semenescu et al. reported the content of the following flavonoids: apigenin (1), epicatechin (9), isoquercitrin (16), luteolin (18), and rutin (21), as well as the following phenolic acids: chlorogenic acid (23), p-coumaric acid (25), ferulic acid (26) [84]. The comparative approach of the study suggests that *G*. *verum* extracts possess promising antioxidant, antimicrobial, and anticancer properties, which strongly depend on the extraction method. The diethyl ether extract demonstrated the highest bioactivity, particularly in terms of antioxidant capacity, antibacterial effects against Gram-positive bacteria, and cytotoxicity against malignant melanoma cells. These findings highlight the importance of extraction methods in optimizing the therapeutic potential of *G*. *verum* for future biomedical applications.

#### 3.2.2. Content of Iridoid Glycosides

Iridoids are a large group of naturally occurring compounds found in medicinal plants, known for their broad range of pharmacological effects [109,110,111,112]. The plants of the genus *Galium* have long been recognized for their rich iridoid content. Research on iridoids began in 1978, when Corrigan et al. studied 12 species of the genus and discovered that they all contained similar iridoids, supporting the idea of a close relationship between species within the genus, which remains accepted today [43]. A few years later, Bojthe-Horvath et al. isolated tricyclic iridoid glycosides from the aerial parts of *G. verum* using spectroscopic and X-ray diffraction methods, marking the first X-ray analysis of such compounds [44,45]. Later research has further supported and expanded these findings, as scientists have used more advanced analysis methods, such as spectrophotometry and liquid and gas chromatography [33,46,47].

Mitova et al. studied 19 species of the genus *Galium* in Bulgaria and used the content of specific iridoid acids to suggest that the iridoid composition of the genus’s ancient ancestors underwent early evolutionary changes [46]. These changes led to the formation of three distinct evolutionary lines, each with a specific phytochemical composition. These lines contributed to the differentiation of the species we know today. One of these lines, including *G. verum*, *G. mollugo*, and *G. humifusum*, resulted in distinct iridoid content, such as iridoid esters, hydroxy and carboxy derivatives of iridoids (like asperuloside), and secoiridoids. The study confirmed a close relationship between *G. verum* and *G. humifusum*, which share similar chemical profiles and likely evolved from a common ancestor.

Demirezer et al. employed spectrophotometric and chromatographic methods to isolate seven types of iridoids from the phytochemical composition of *G. verum*, including asperuloside (28), asperulosidic acid (29), daphylloside (30), deacetyl-asperulosidic acid (31), and monotropein (35) [47].

In a study of *G. verum* var. *asiaticum* in Korea, seven derivatives of iridoid glycosides were reported: asperuloside (28), asperulosidic acid (29), deacetylasperuloside (32), and scandoside (36) [48].

Friščić et al. researched eight species of the genus *Galium* and reported that *G. verum* has the highest iridoid content among them, confirming previous studies [33]. The study found that asperuloside (28) was the most common iridoid. Asperuloside is an iridoid glucoside, which gives a positive blue reaction when tested with the Trim-Hill reagent [113]. The study also indicated that the highest yield of asperuloside is obtained when the plants are fully in bloom, as the inflorescences contain the highest iridoid content. According to the study, although iridoids dominate the composition of *G. verum*, no correlation was found between their quantity and the plant’s strong antiradical capacity.

Camero et al. investigated the antiangiogenic effects of iridoids isolated from *G. tunetanum*, some of which are also present in *G. verum*, such as asperuloside (28), asperulosidic acid (29), daphylloside (30), deacetyl-asperuloside (32), geniposidic acid (33), and monotropein (35) [49]. The study applied an in vivo model using the chorioallantoic membrane of a hen’s egg with a developed embryo. The iridoids isolated from *G. tunetanum* were applied to the membrane, resulting in the inhibition of microvessel formation compared to the control. The antiangiogenic effect, expressed as a percentage of inhibition compared to the control, reached 67%. This inhibition of angiogenesis could potentially be a promising approach for anticancer therapies.

A study of species in the genus *Galium*, including *G. verum*, conducted in Estonia by Laanet et al., found similar levels of asperulosidic acid (29), which confirmed the thesis on the chemotaxonomic role of iridoids [37]. Hydroacetone extract yielded higher amounts of asperuloside (28), although the hydroethanol extract, which produced slightly lower yields, is considered more suitable for biological applications.

Numerous pharmacological studies confirmed that naturally occurring iridoids in *G. verum* have a wide range of beneficial properties that can be utilized for the therapy and prevention of various diseases. Iridoids, especially asperuloside, can exhibit the following properties: neuroprotective, immunomodulatory, antidiabetic, cardioprotective, antihepatotoxic, hepatoprotective, choleretic, hypoglycemic, hypolipidemic, anti-inflammatory, antispasmodic, antitumor, antiviral, antibacterial, and antifungal [102,103]. Asperuloside has been identified as a key compound responsible for the sedative effects observed in plants of the genus *Galium*. Based on this evidence, Bradic et al. suggest that *G. verum* may be beneficial for managing nervousness and phobias [50]. In addition, iridoids extracted from *Galium* species also exhibit antiangiogenic properties by suppressing the formation of new blood vessels that support tumor growth [38,49]. This makes *Galium* species suitable for application in cancer treatment.

#### 3.2.3. Content of Anthraquinones

Anthraquinones are phytochemicals with notable biological activity that have the ability to affect some physiological processes at the cellular level. Thus, they can exhibit antimicrobial effects on some pathogens and induce apoptosis in cancer cells [46,54,75,76,114,115]. However, their manner of action must be fully elucidated as they exhibit cytotoxicity not only on cancer cells but also on normal ones [65,66].

Studying *G. mollugo* cells, Heide et al. reported that anthraquinones are located in vacuoles and are present in small amounts [51]. This explains the limited data on these compounds in members of the genus *Galium*.

As early as 1995, Banthorpe et al. [52] were among the first to report information about the identification of anthraquinones in *G. verum.* Zhao et al. [53] confirmed the content of anthraquinones, including physcione (37) and rubiadin (38). Physcion was isolated for the first time from the genus *Galium*. Kanso et al. found that anthraquinones are more abundant in the roots of *G. verum* than in other parts of the plant [54]. The study also highlighted the biological effects of these compounds, such as their antibacterial and anticancer potential. This activity was explained by the inhibitory effect of anthraquinones on nucleic acid synthesis in bacterial cells and their induced destruction [55]. Anthraquinones are highly polar, which gives them a stronger bactericidal effect against drug-resistant bacteria, including both Gram-positive and Gram-negative bacteria.

#### 3.2.4. Content of Terpenoids (Monoterpenoids, Sesquiterpenoids, Triterpenoids, Triterpenoid Saponins) and Volatile Organic Compounds (VOCs)

Terpenoids are among the largest classes of secondary metabolites and are produced in plants, playing a significant role in pharmacology and plant defense along with VOCs. Triterpenoid saponins are known for their broad antifungal and antimicrobial potential and also exhibit a wide range of biological activities, such as anticancer and anti-inflammatory effects. The antimicrobial properties of some species of the genus *Galium* are attributed to the interaction between sterols on bacterial erythrocyte membranes and saponins [116].

In 2006, monoterpene glycosides were reported for the first time in the composition of *G. verum*. The identified compounds were included in betulalbuside A (40) [47]. Later, Ilyina et al. conducted a study on essential oil from flowers of *G. verum* using the GC-MS technique [56]. The detected lipophilic volatile compounds included α-Terpineol (39), borneol (42), camphor (43), squalene (49), and some unknown substances. Phytochemical screening conducted by Friščić et al. confirmed that *G. verum* contains not only flavonoids, tannins, and iridoids but also saponins and triterpenes [33].

Shynkovenko et al. determined the content of triterpene saponins in *G. verum* by the HPLC method [57]. The saponins identified include the ursane type: euscaphic acid (44), tormentic acid (50), ursolic acid (51), and uvaol (52); the oleanane type: oleanolic acid (47); and the lupane type: botulin (41) and lupeol (46). Tava et al. identified a total of more than 70 volatile compounds in the composition of *G. verum* [58]. The largest amounts were detected of germacrene D (45) in the flowers. According to the study, the specific phytochemical composition has a significant ecological role, as it protects the plants from herbivores and aphids in their environment and attracts pollinators.

Ciotlaus et al. conducted a comparative study on the content of volatile organic compounds in fresh and dried *G. verum* plants [59]. The fresh aerial parts contained a total of twenty-eight compounds, while the dried flower contained a total of fifty compounds. The fresh floral bouquet comprised mainly oxygenated monoterpenes, while the dried bouquet contained mainly aldehydes, monoterpenes, alcohols, sesquiterpenes, and acetates.

#### 3.2.5. Phytosterol Content

Phytosterols are an important part of a person’s diet, not only because they help reduce blood cholesterol but also because they have been shown to exhibit antitumor activity through several mechanisms: by inducing apoptosis, enhancing immune recognition of cancer cells, and directly inhibiting tumor growth [117,118].

Research on phytosterols in *G. verum* is limited. One of the few studies published on this topic is by Qing-jie et al. [61]. The researchers analyzed the chemical composition of *G. verum* via GC-MS. Some of the components were identified for the first time in *G. verum*. The results showed the presence of β-sitosterol (53), campesterol (54), and stigmasterol (55).

Mocan et al. studied four species of the genus *Galium* and identified two phytosterols, β-sitosterol (53) and campesterol (54), in all of them [62]. The determined amounts were 85.46 ± 1.24 μg/g and 9.86 ± 0.04 μg/g, respectively. This study offers a scientific basis for the traditional uses of *Galium* species because these secondary metabolites might be responsible for the pharmacological effects of *Galium* plants.

Since existing data suggest that the intake of phytosterols, such as stigmasterol and β-sitosterol, can reduce the risk of tumor development, it would be useful to study the mechanism of action of phytosterols found in *G. verum*.

#### 3.2.6. Pharmacological Effects of *G. verum*

The pharmacological properties exhibited by species of the genus *Galium* on biological systems are mainly due to their high antioxidant activity. Integrating antioxidant therapy is of most importance to enhance the safety and efficacy of conventional treatments [97]. The radical scavenging potential [26], antiangiogenic effects [38], and cytotoxicity [65,66] of *Galium* plants contribute to their anticancer effects. Various studies have shown that flavonoids are of leading importance for the anticancer effect of *G. verum*. In two consecutive years, Schmidt et al. conducted an in vitro experiment and proved that the decoction of *G. verum* exhibited cytotoxicity and inhibited cancer cell growth. Additionally, the decoction protected the DNA of epithelial cells from primary mucosa against the mutagenic effects of benzo[a]pyrene, a key DNA-damaging agent found in cigarette smoke. These findings support the idea that *G. verum* could be a valuable source for developing herbal medicines aimed at cancer prevention and therapy [65,66].

Khalili et al. determined the antihaemolytic activity of plant extracts of ten known for their antioxidant activity plants [67]. Antihaemolytic activity was examined in the red blood cells of mice. Vitamin C has been selected as a control antioxidant. Nine of the extracts were more potent than vitamin C, of which *G. verum* was the most potent. Hemolysis inhibition was related to the method of extraction and extract concentration. This study proved that *G. verum* could be used as a source of natural antioxidants for the pharmaceutical industry.

The hepatoprotective activity of the extract of *G. verum* was tested on carbon tetrachloride-induced acute hepatitis in rats [68]. The treatment with plant extracts led to the improvement of all assessed biochemical parameters and histopathological analysis. The hepatoprotective effect was comparable with the hepatoprotective activity of the reference drug.

A comparative study of the diuretic activity of the aqueous and alcoholic extracts of *G. verum* was conducted [69]. The extracts were applied to rats according to Berkhin’s method [119]. The results demonstrated that a 60% ethanol extract of *G. verum* exhibits the most pronounced diuretic effect. According to this study, the diuretic activity of *G. verum* is based on flavonoids, anthracene derivatives, and terpenoid content.

A series of rat experiments were conducted from 2019 to 2023 to evaluate the biological properties of the methanolic extract of *G. verum* [70,71,72]. The effects of the methanol extract of *G. verum* were investigated on the redox status of isolated hearts of spontaneously hypertensive rats after ischemia [70]. The results showed that 4-week treatment has the ability to significantly alleviate cardiac oxidative stress in a dose-dependent manner. Other researchers tested the effects of this extract on myocardial ischemia in spontaneously hypertensive rats, too [71]. After four weeks of treatment, it was found that the methanol extract had positive effects on heart function as it decreased the generation of pro-oxidants, thus reducing oxidative damage. In another study, rats were treated with doxorubicin-induced cardiotoxicity, also by the methanol extract of *G. verum* [72]. The extract was proven to increase the activity of the antioxidant defense system and prevent the pathological injuries caused by doxorubicin via a decrease in oxidative stress and apoptosis. The results of these studies overlap and confirm the high cytoprotective effect of methanol extracts prepared from *G. verum*. However, more data are needed to fully clarify the mechanism of action of the extracts of *G. verum*.

Another study demonstrated that the methanol extract of *G. verum* exhibits a cytotoxic effect when applied to colon cancer cells (HT29) [75]. As a result, the level of reactive oxygen species (ROS) increases in the mitochondria of the cancer cells, leading to the induction of apoptosis. Apoptosis is a process of programmed cell death, which occurs naturally and is beneficial to the body, especially in response to changes in environmental factors or the development of tumor cells. When the DNA of these tumor cells is damaged, they stop dividing and eventually die. This study explains the mechanism by which phenolic compounds in *G. verum* work, revealing their therapeutic potential for treating tumor-related diseases.

The studies conducted by Pashapour et al. found a connection between the dose of plant extract and the occurrence of side effects in non-tumor human cells [76]. At high doses above 400 μg/mL, there was an increased induction of apoptosis in normal cells, which decreased their survival rate.

The research of Marković et al. on the ethnobotanical use of plants from the genus *Galium* used an unusual approach [77]. It was performed as a survey among the rural population in four municipalities of Pirot District in Serbia. The people interviewed claimed that infusion from *G. verum* can be used for the treatment of inflammation of the oral cavity, hoarseness, kidney and bladder diseases, diabetes, and fainting, while compression can be applied for skin diseases and irritation.

Other studies conducted using the same method show that the plant has been used for its sedative and wound-healing action and as an enzyme activator [79,80,81].

### 3.3. Relationship Between Bioactive Compounds, Biological Activities, and Medicinal Uses

*Galium* species contain a diverse range of phytochemical compounds, contributing to their wide array of potential applications in medicine and health. Their potential is primarily due to the multiple mechanisms of action they possess. This makes them suitable for therapy and the prevention of various diseases.

These compounds include flavonoids, phenolic acids, iridoids, anthraquinones, saponins, and essential oils. They exhibit significant biological activity and exert numerous beneficial effects on various human body systems, including the central nervous, hepatobiliary, renal, gastrointestinal, and urinary systems. Their therapeutic potential is largely attributed to their preventive actions, including antioxidant, antibacterial, antifungal, anticancer, and immunomodulatory properties.

Polyphenols and flavonoids are known for their strong antioxidant properties. Flavonoids such as quercetin, rutin, kaempferol, and luteolin have the ability to increase reactive oxygen species (ROS) in mitochondria, damaging cellular DNA, which leads to the induction of apoptosis in cancer cells [75,76,94,95,120]. Flavonoids can also exhibit pro-oxidant activity under certain reaction conditions [96]. Their anti-inflammatory properties stem from their ability to inhibit pro-inflammatory cytokines, making them relevant for conditions such as arthritis and cardiovascular diseases [95].

Polyphenols include phenolic acids such as chlorogenic acid, caffeic acid, ferulic acid, and gallic acid. They have the ability to neutralize radicals, regulate nitric oxide, and inhibit cell proliferation [20,32,38,59,99,100]. In addition to antioxidant properties, these compounds also exhibit antimicrobial [23] and hepatoprotective properties [68], making them effective in preventing liver damage and bacterial infections.

Iridoids—asperuloside, loganin, geniposidic acid, and scandoside—may exhibit immunomodulatory, antidiabetic, hepatoprotective, choleretic, neuroprotective, anti-inflammatory, and antibacterial effects, though their exact mechanisms are still not fully understood [68,92]. Asperuloside has been shown to exhibit sedative and antiangiogenic effects in vitro [38,111,112], and loganin has been linked to the reduction in neuroinflammation, which is potentially beneficial for Alzheimer’s disease [112].

Anthraquinones, such as rubiadin and physcion, are known for their antimicrobial, anticancer, anti-osteoporotic, hepatoprotective, and neuroprotective activities, making them promising candidates for drug development [121]. They can destroy bacterial cell walls, inhibit nucleic acid and protein synthesis, and interfere with bacterial respiration, leading to a bacteriostatic effect. Furthermore, due to their high polarity, anthraquinones possess antibacterial activity against drug-resistant bacteria, including both Gram-positive and Gram-negative bacteria [109,114,115].

Saponins (betalbuside A), along with other terpenoids (germacrene D) and volatile organic compounds (squalene) from the content of essential oils of *Galium* species, possess antifungal, antiviral, antimicrobial, and antitumor effects, making them useful for application on skin and respiratory infections [32,54,56,58,59].

The high phytosterol content in *Galium* species contributes to their anticancer activity, as phytosterols promote apoptosis in cancer cells, leading to programmed cell death [54,118,122].

**Figure 1:**
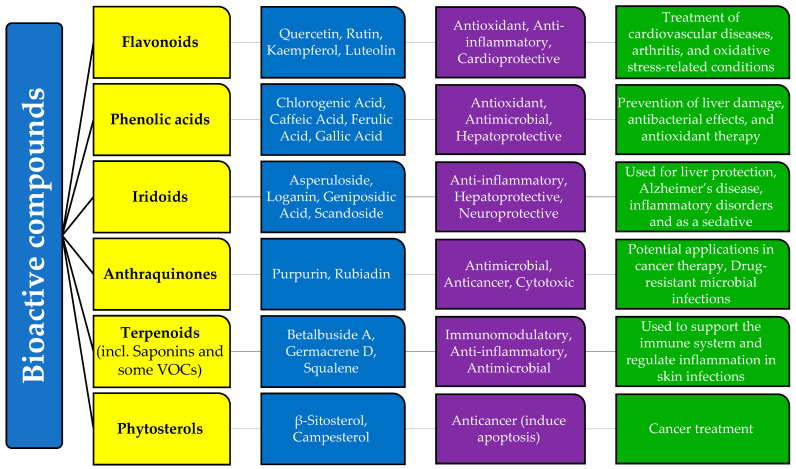
Relationship between bioactive compounds, biological activities, and medicinal uses of *Galium* species (Rubiaceae)/*Galium verum* L. according to the scientific research.

Many bioactive compounds found in *G. verum,* such as rutin, kaempferol, quercetin, luteolin, chlorogenic acid, caffeic acid, scandoside, asperuloside, β-sitosterol, and campesterol, have also been reported in other species of the genus and the Rubiaceae family, suggesting a biochemical consistency that may lead to similar pharmacological effects. Flavonoids including quercetin, rutin, and kaempferol are common in other Rubiaceae genera, such as *Morinda* and *Coffea*, where they exert similar antioxidant and anti-inflammatory properties [6]. Iridoids, including asperuloside and loganin, are reported in Morinda citrifolia, where they exhibit neuroprotective effects similar to those in *Galium* species [6]. Anthraquinones, such as rubiadin, are present in Rubia cordifolia, another Rubiaceae plant known for its antimicrobial and anticancer properties [4]. Saponins identified in *G. verum* are structurally related to saponins found in other medicinal plants used for immune modulation [6,56,57]. Figure 1 represents the summarized results of our research.

## 4. Relevance of the Topic and Current Research Trends

**Figure 2:**
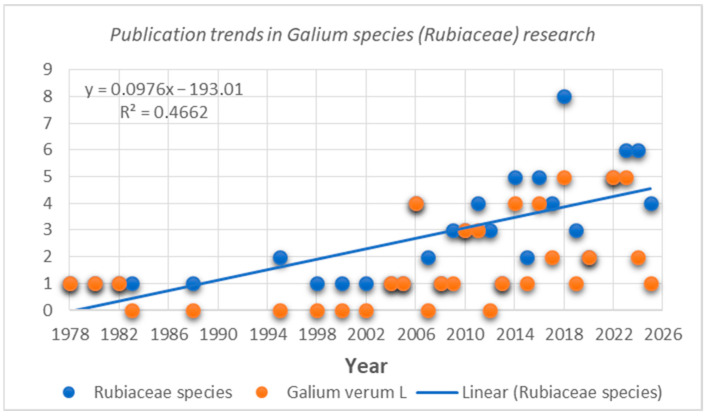
Publication trends in research connected with *Galium* species (Rubiaceae)/*Galium verum* L.

The data presented in this review have been carefully selected and organized to provide a comprehensive overview of the phytochemical constituents and biological effects of *G. verum* and the *Galium* genus as a whole, helping to obtain a clear understanding of the potential they offer. This literature review discusses a total of 83 scientific studies related to the composition and properties of *Galium* species (*Rubiaceae*). Notably, 60% of the published research focuses on *G. verum*, highlighting the strong interest in this species. The number of studies has increased significantly since 1978, with 30% of scientific studies being published after 2020 (Figure 2).

## 5. Conclusions

This review covers phytochemical studies of *Galium* species for the past 47 years (1978–2025). The reported bioactive compounds have been isolated mainly from *G.verum*, *G. aparine*, *G. mollugo*, and *G. odoratum.* Based on the information collected, this review shows that plants of the genus *Galium*, including *G. verum*, are rich in diverse chemical compounds with strong biological activity. Most studies focus on the phenolic content of the plant extracts and their associated antioxidant activity. The increasing interest in biologically active compounds from *Galium* species reflects the growing recognition of their pharmacological potential. The topic remains highly relevant, as recent studies continue to explore their diverse phytochemical composition and therapeutic applications. Many of the bioactive compounds identified in *Galium* species, such as flavonoids, iridoids, phenolic acids, anthraquinones, and saponins, are also found in other members of the genus and the broader Rubiaceae family. These compounds have already been investigated for their medicinal properties and have demonstrated significant pharmacological effects, including antioxidant, anti-inflammatory, antimicrobial, hepatoprotective, and anticancer activities. Their established therapeutic applications further support the relevance of *Galium* species in modern medicine, highlighting the need for continued research to fully elucidate their mechanisms of action and clinical potential.

## 6. Future Directions

The scientific research on *Galium* species, particularly *G. verum*, supports their traditional medicinal applications. However, comprehensive studies on their full pharmacological potential remain limited. Future research should prioritize the clinical validation of isolated bioactive compounds to establish their efficacy and safety for therapeutic use. Additionally, further studies should explore the mechanisms of action, pharmacokinetics, and potential synergistic effects between different compounds. Expanding research to include large-scale in vivo and clinical trials will be essential in determining their role in modern medicine. Investigating new drug formulations, bioavailability improvements, and personalized medicine applications may also enhance their clinical relevance. Moreover, optimizing and standardizing extraction methods is crucial to ensuring the efficient isolation of bioactive compounds. Comparative studies on solvent selection, extraction efficiency, and compound stability should be conducted to determine the most effective techniques for preserving the pharmacological properties of *Galium* species. Additionally, future research should examine the influence of geographical and environmental factors on the phytochemical composition of *Galium* species, as variations in climate, soil composition, and altitude may significantly affect the concentration and diversity of bioactive compounds. Furthermore, further research to develop antioxidant-based therapies should be explored to enhance their potential in preventing and managing oxidative stress-related diseases.

## Figures and Tables

**Table 1:** Phytochemistry of *Galium* species.

Species	Phytochemical Group of Compounds	Compounds	Reference
*G. aparine*	Flavonoids	Astragalin; Hyperoside; Isorhamnetin 3-O-rhamnoglucoside; Isorhamnetin-3-O-rutinoside; Isoquercitrin; Kaempferol; Kaempferol-3-O-β-D-glucopyranosyl-7-O-α-L-rhamnopyranoside; Kaempferol-7-O-α-L-rhamnopyranoside; Luteolin; Nicotiflorin; Quercetin; Quercetin glycoside; Quercetin-3-O-β-D-glucopyranoside; Rutin	[23,28,37,42,54]
	Polyphenols	Caffeic acid; Chlorogenic acid; Coumaric acid; Cryptochlorogenic acid; Dicaffeoylquinic acid isomer; Ferulic acid; Gallic acid; Neochlorogenic acid; p-Coumaric acid; p-Hydroxybenzoic acid; Protocatechuic acid	[23,37,41]
	Iridoids	10-Desacetylasperulosidic acid; Asperuloside; Asperulosidic acid; Deacetylasperulosidic acid; Geniposide; Monotropein; Scandoside	[37,41,42,46,54]
	Anthraquinones	Emodin; Rhein	[41,54]
	Terpenoids	Betulin; Euscaphic acid; Lupeol; Oleanolic acid; Tormentic acid; Ursolic acid	[57]
	Phytosterols	β-Sitosterol; Campesterol	[62]
*G. mollugo*	Flavonoids	Astragalin; Catechin; Cosmosiin; Cynaroside; Diosmetin; Diosmetin 7-O-β-D-glucopyranoside; Diosmetin 7-O-β-D-xylopyranosyl-1,6-β-D-glucopyranoside; Diosmetin isomer; Epicatechin; Hesperidin; Hispidulin; Hyperozide; Isorhamnetin; Isorhamnetin 3-O-α-L-rhamnopyranosyl-1,6-β D-glucopyranoside; Isorhoifolin; Kaempferol; Kaempferol-O-glucoside; Luteolin; Quercetin; Quercetin-3-O-β-D-glucopyranoside; Quercitrin; Rutin	[23,37,50,54]
	Polyphenols	Caffeic acid; Chlorogenic acid; Complicatus; Cryptochlorogenic acid; Ferulic acid; Gallic acid; p-Coumaric acid; Coumaric acid	[23,28,37,50,54]
	Iridoids	10-Desacetyl asperulosidic acid; 10-Hydroxyloganin; 10-Hydroxymorroniside; Asperulosidic acid; Asperuloside; Deacetylalpinoside; Deacetyl-daphylloside; Deacetylasperulosidic acid; Daphylloside; Galioside; Gardenosidic acid; Geniposidic acid; Loganin; Mollugoside; Monotropein; Scandoside; Scandoside methyl ester; Secogalioside; 6-Acetylscandoside; 6-O-epi-Acetyl scandosid	[37,42,50,54]
	Phytosterols	β-Sitosterol; Campesterol	[62]
*G. odoratum*	Flavonoids	Kaempferol; Quercetin; Quercitrin; Rutin	[23,40]
	Polyphenols	Caffeic acid; Chlorogenic acid; p-Coumaric acid; Ferulic acid	[23,40,54]
	Iridoids	10-Deacetyl asperulosidic acid; Asperulosidic acid; Asperuloside; Deacetyl asperuloside; Geniposidic acid; Monotropein; Scandoside	[40,46,54]
	Phytosterols	β-Sitosterol; Campesterol	[54,62]
*G. verum*	Flavonoids	Apigenin; Apigetrin; Astragalin; Catechin; Chrysin; Cynaroside; Diosmin; Diosmetin; Epicatechin; Fisetin; Hesperidin; Hyperoside; Isorhoifolin; Isorhamnetin; Isoquercetin; Isoquercitrin; Kaempferol; Luteolin; Quercetin; Quercitrin; Rutin	[16,17,18,20,21,22,23,25,28,29,35,36,37,47,48,50,52,54,84]
	Polyphenols	Caffeic acid; Chlorogenic acid; Coumarinic acid; p-Coumaric acid; Ferulic acid; Gallic acid;	[16,20,22,23,25,35,37,50,84]
	Iridoids	Asperuloside; Asperulosidic acid; Daphylloside; Deacetyl-asperulosidic acid; Deacetyl-asperuloside; Geniposidic acid; Loganin; Monotropein; Scandoside	[37,46,50,54]
	Anthraquinones	Physcion; 1,3-Dihydroxy-2-methylanthraquinone (Rubiadin)	[53]
	Terpenoids	α-Terpineol; Betulalbuside A; Betulin; Borneol; Camphor; Euscaphic acid; Germacrene D; Lupeol; Oleanolic acid; Rubifolic acid; Squalene; Tormentic acid; Ursolic acid; Uvaol	[54,57]
	Phytosterols	β-Sitosterol; Campesterol; Stigmasterol	[61,62]

**Table 2:** Chemical structure and biological activity of phytochemicals isolated from *Galium verum*.

№	Phytochemical Compound	Molecular Formula	2D Chemical Structure *	Biological Activity	Ref.
	Flavonoids				
1	Apigenin	C_15_H_10_O_5_	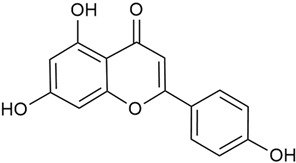	Antioxidant	[22,36,84]
2	Apigetrin (Cosmosiin, Apigenin 7-glucoside)	C_21_H_20_O_10_	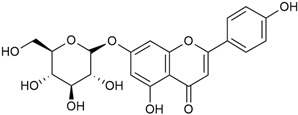		[22,28]
3	Astragalin (Kaempferol 3-O-glucoside)	C_21_H_20_O_11_	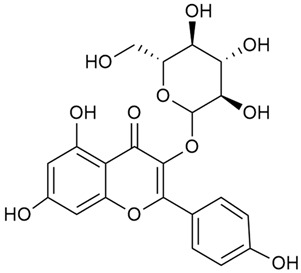		[47]
4	(+)-Catechin (Cianidanol)	C_15_H_14_O_6_	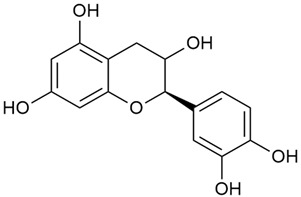	Antioxidant Anti-inflammatory Antimicrobial	[25]
5	Chrysin	C_15_H_10_O_4_	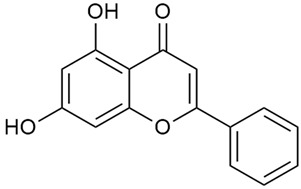		[25]
6	Cynaroside (Luteolin 7-O-glucoside)	C_21_H_20_O_11_	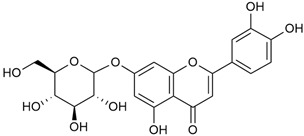		[28]
7	Diosmin	C_28_H_32_O_15_	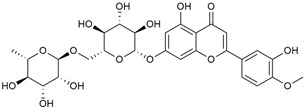	Anti-thrombotic	[29]
8	Diosmetin	C_16_H_12_O_6_	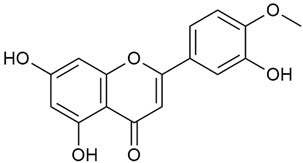	Anticancer Antiapoptotic	[17,18,21]
9	(-)-Epicatechin	C_15_H_14_O_6_	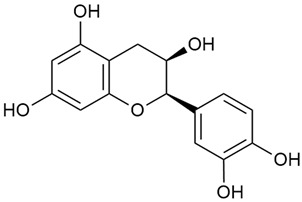		[25,84]
10	Fisetin	C_15_H_10_O_6_	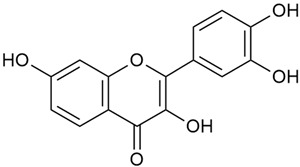		[25]
11	Hesperidin	C_28_H_34_O_15_	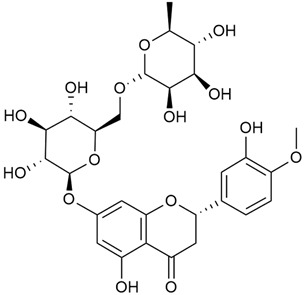		[25]
12	Hyperoside	C_21_H_20_O_12_	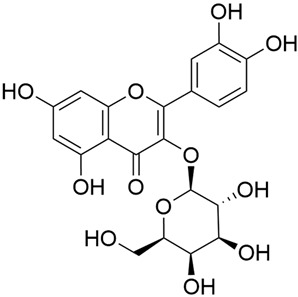		[16,36]
13	Isorhoifolin (Apigenin 7-O-rutinoside)	C_27_H_30_O_14_	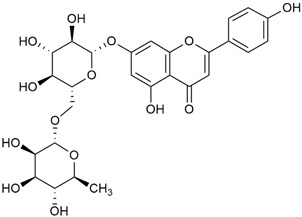		[28]
14	Isorhamnetin	C_16_H_12_O_7_	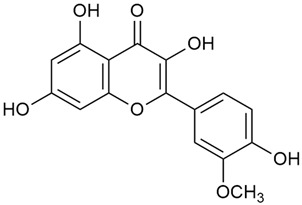		[18]
15	Isoquercetin	C_21_H_20_O_12_	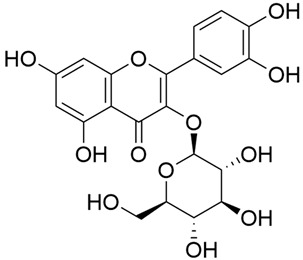		[36]
16	Isoquercitrin	C_21_H_20_O_12_	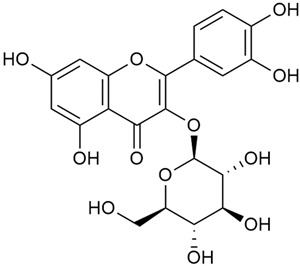	Anticancer	[23,25,84]
17	Kaempferol	C_15_H_10_O_6_	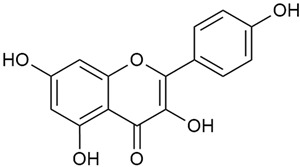	Antioxidant	[22,23]
18	Luteolin	C_15_H_10_O_6_	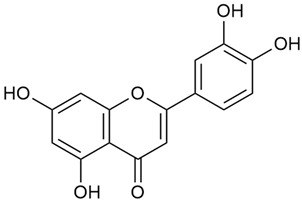	Antioxidant	[20,22,84]
19	Quercetin	C_15_H_10_O_7_	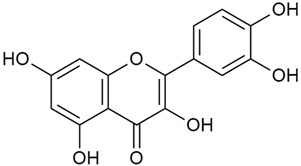	Anti-hypertensive Anti-inflammatory	[22,35,36]
20	Quercitrin	C_21_H_20_O_11_	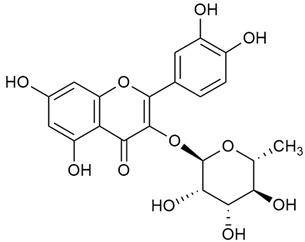	Antioxidant Antimicrobial	[16,23,35,36]
21	Rutin	C_27_H_30_O_16_	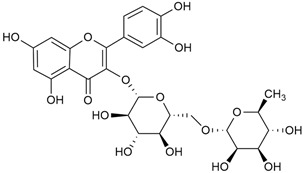	Antioxidant Anticancer Antimicrobial	[16,20,22,35,36,47,84]
	Phenolic acids				
22	Caffeic acid	C_9_H_8_O_4_	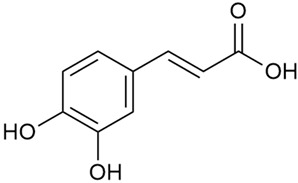	Antioxidant	[16,20,22,25]
23	Chlorogenic acid	C_16_H_18_O_9_	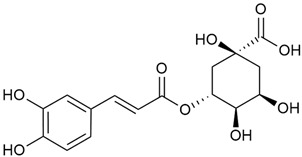	Antioxidant Anticancer Antimicrobial	[16,22,23,25,35]
24	Coumarinic acid	C_9_H_8_O_3_	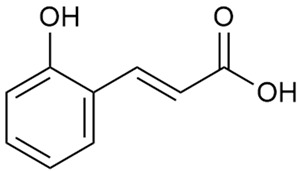		[22]
25	p-Coumaric acid	C_9_H_8_O_3_	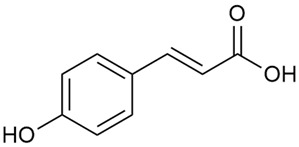	Antioxidant	[22,23,25,35,84]
26	Ferulic acid	C_10_H_10_O_4_	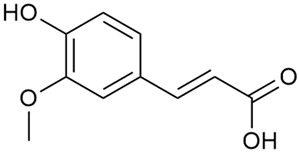		[22,23,25,84]
27	Gallic acid	C_7_H_6_O_5_	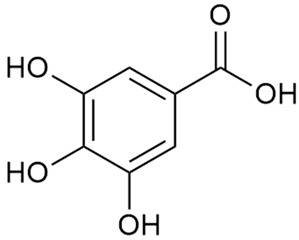		[35]
	Iridoid glycosides				
28	Asperuloside	C_18_H_22_O_11_	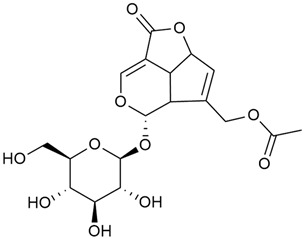	Antioxidant Anticancer Anti-inflammatory	[46,47,48]
29	Asperulosidic acid	C_18_H_24_O_12_	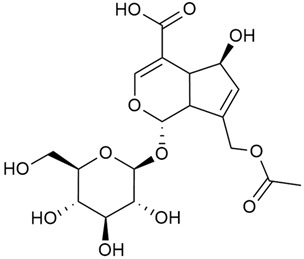		[46,47,48]
30	Daphylloside	C_19_H_26_O_12_	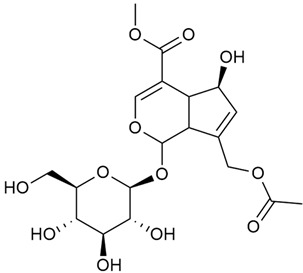	Hepatoprotective	[47]
31	Deacetylasperulosidic acid	C_16_H_22_O_11_	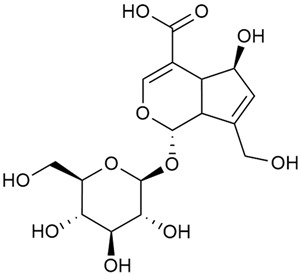		[46,47]
32	Deacetylasperuloside	C_16_H_20_O_10_	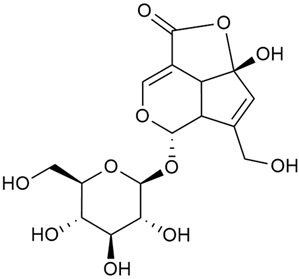		[48]
33	Geniposidic acid	C_16_H_22_O_10_	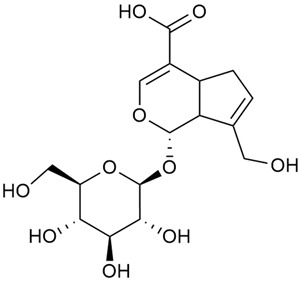		[44,45]
34	Loganin	C_17_H_26_O_10_	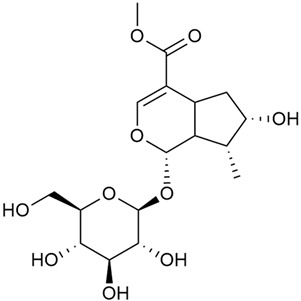		[46]
35	Monotropein	C_16_H_22_O_11_	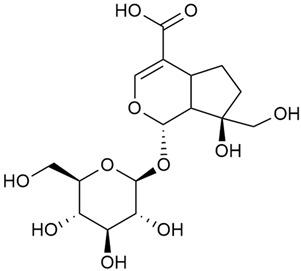		[46,47]
36	Scandoside	C_16_H_22_O_11_	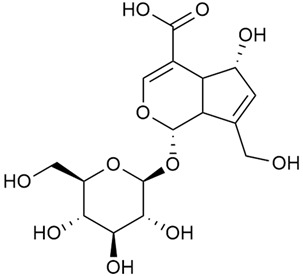		[46,48]
	Anthraquinones				
37	Physcion	C_16_H_12_O_5_	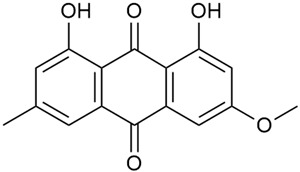		[53]
38	Rubiadin (1,3-dihydroxy-2-methylanthraquinone)	C_16_H_12_O_5_	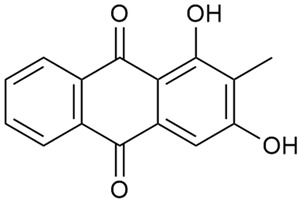		[53]
	Terpenoids				
39	α-Terpineol	C_10_H_18_O	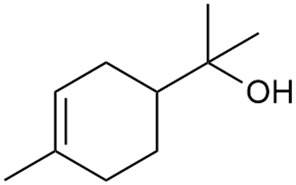		[56,58]
40	Betulalbuside A	C_16_H_28_O_7_	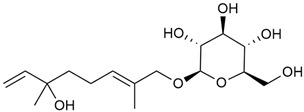		[47]
41	Betulin	C_30_H_50_O_2_	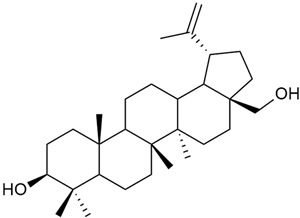		[57]
42	Borneol	C_10_H_18_O	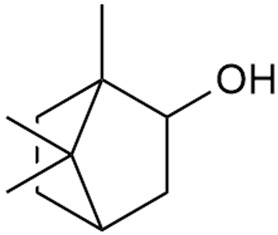		[56,58]
43	Camphor	C_10_H_16_O	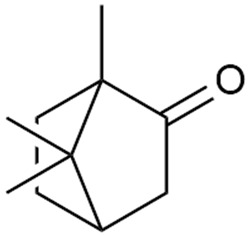	Antioxidant Cytotoxic Antimicrobial	[56,59]
44	Euscaphic acid	C_30_H_48_O_5_	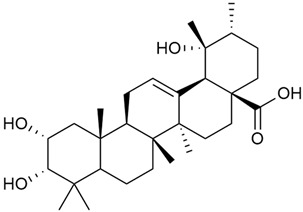		[57]
45	Germacrene D	C_15_H_24_	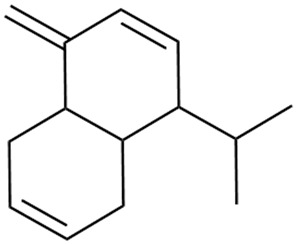	Antioxidant Antimicrobial	[58]
46	Lupeol	C_30_H_50_O	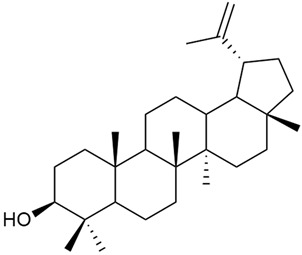		[57]
47	Oleanolic acid	C_30_H_48_O_3_	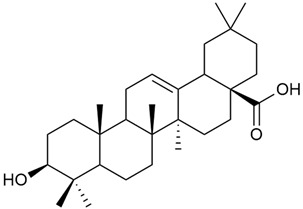		[57]
48	Rubifolic acid	C_30_H_48_O_4_	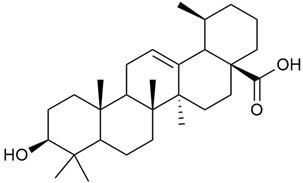		[18,32]
49	Squalene	C_30_H_50_	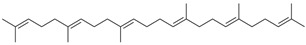	Antioxidant Cytotoxic Antimicrobial	[56,58,59]
50	Tormentic acid	C_30_H_48_O_5_	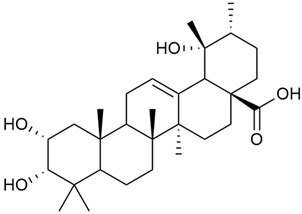		[57]
51	Ursolic acid	C_30_H_48_O_3_	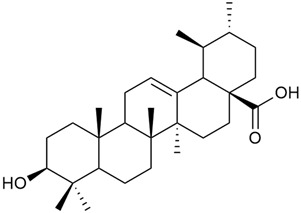		[18,32,57]
52	Uvaol	C_30_H_50_O_2_	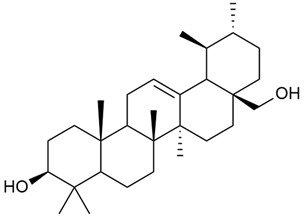		[57]
	Phytosterols				
53	β-sitosterol	C_29_H_50_O	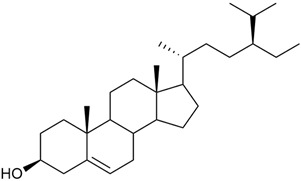	Antioxidant Anticancer Anti-inflammatory	[62]
54	Campesterol	C_28_H_48_O	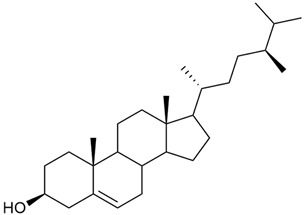	Anticancer	[61,62]
55	Stigmasterol	C_29_H_48_O	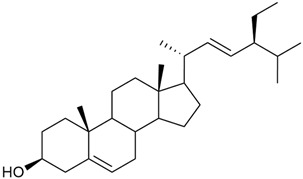		[61]

* Two-dimensional chemical structures, drawn with ChemDraw Software.

## Data Availability

Not applicable.
